# Estimating core body temperature using electrocardiogram signals

**DOI:** 10.1371/journal.pone.0270626

**Published:** 2022-06-28

**Authors:** Chie Kurosaka, Takashi Maruyama, Shimpei Yamada, Yuriko Hachiya, Yoichi Ueta, Toshiaki Higashi

**Affiliations:** 1 Department of Human, Information and Life Sciences, School of Health Sciences, University of Occupational and Environmental Health, Kitakyushu, Japan; 2 Department of Physiology, School of Medicine, University of Occupational and Environmental Health, Kitakyushu, Japan; 3 Department of Occupational Safety and Health Management, School of Health Sciences, University of Occupational and Environmental Health, Kitakyushu, Japan; 4 Department of Occupational and Community Health Nursing, School of Health Sciences, University of Occupational and Environmental Health, Kitakyushu, Japan; 5 Nishinihon Occupational Health Service Center, Kitakyushu, Japan; University of Illinois at Urbana-Champaign, UNITED STATES

## Abstract

Suppressing the elevation in core body temperature is an important factor in preventing heatstroke. However, there is still no non-invasive method to sense core body temperature. This study proposed an algorithm that estimates core body temperature based on electrocardiogram signals. A total of 12 healthy men (mean age ± SD = 39.6 ± 13.4) performed an ergometric exercise load test under two conditions of exercise load in an environmental chamber adjusted to a temperature of 35°C and humidity of 50%. Vital sensing data such as electrocardiograms, core body temperatures, and body surface temperatures were continuously measured, and physical data such as body weight were obtained from participants pre- and post-experiment. According to basic physiological knowledge, heart rate and body temperature are closely related. We analyzed the relationship between core body temperature and several indexes obtained from electrocardiograms and found that the amount of change in core body temperature had a strong relationship with analyzed data from electrocardiograms. Based on these findings, we developed the amount of change in core body temperature estimation model using multiple regression analysis including the Poincaré plot index of the ECG R-R interval. The estimation model showed an average estimation error of -0.007°C (average error rate = -0.02%) and an error range of 0.457–0.445°C. It is suggested that continuous core body temperature change can be estimated using electrocardiogram signals regardless of individual characteristics such as age and physique. Based on this applicable estimation model, we plan to enhance estimation accuracy and further verify efficacy by considering clothing and environmental conditions.

## Introduction

Heatstroke has become more prevalent in recent years [1, 2], thus constituting a social problem [3]. Heatstroke is defined as a failure of the thermoregulatory system of the central nervous system and results in multi-organ damage and failure due to elevated temperatures [4]. Pathophysiological processes of heatstroke at the cellular level include an inflammatory reaction, and multi-organ failure due to the combination of high body temperature and circulatory disorder may eventually lead to lethal disseminated intravascular coagulation, defined “clinically as a core body temperature that rises above 40°C and that is accompanied by hot, dry skin and central nervous system abnormalities such as delirium, convulsions, or coma” [5]. Countermeasures include changes to outdoor working conditions, altered working hours in business and educational settings, environmental improvements designed to facilitate break times, and educational provisions concerning the nature of the issue itself. As severe cases of heatstroke can be life-threatening, it is important to ensure adequate occupational health management (e.g., water supplementation and rest) in addition to providing health education aimed at raising awareness about appropriate preventive measures. While these elements have proactively been implemented in a variety of contexts, deaths due to heatstroke continue to occur. Adding to the concern, the Intergovernmental Panel on Climate Change [6] reported substantial increases in global average surface temperatures over the past 50 years due to global warming, which has largely been triggered by human activity. As this is a worldwide issue, international efforts are targeted at reducing greenhouse gas emissions, starting with carbon dioxide. However, these improvements will take time, while temperatures are continually expected to rise over the foreseeable future. This makes it urgent to develop and implement technologies that can continuously manage changes in physical conditions when working in hot environments.

When evaluating heat stress, it is important to consider factors related to heat dissipation from the human body—such as, radiative heat (radiation temperature) and wind speed—in addition to measurable environmental factors (e.g., temperature and humidity). These risk factors must be comprehensively evaluated even in relatively cool environments, as the heat transfer rate varies due to exercise intensity and several other points of concern, including the heat retention/thermal insulation properties of clothing, age, and physical condition. The guidelines of the American Conference of Governmental Industrial Hygienists [4] recommend the termination of heat exposure in cases where the heart rate does not return to 120 or less within 1 min after reaching peak work intensity. The ISO 9886 [7] issued four heat risk evaluation indexes for determining work discontinuation, including core body temperature (rectal, esophageal, intraperitoneal, tympanic membrane, ear canal, and urine temperature), skin temperature (local site, multiple site average), heart rate, and body weight reduction; a rectal temperature of 38.0–38.5°C and skin temperature of 43°C (according to heat acclimation) are considered work termination threshold values. In addition, the ISO 9886 also states that the maximum heart rate should not exceed a threshold of 185–0.65×(age) or continuous heart rate of 180–(age).

Body temperature is roughly categorized into brain/internal organ temperature (core body temperature) and skin surface temperature. As elevated core body temperature increases the risk of heatstroke onset, it is critical to measure body temperature at a bodily location as deep inside the body as possible (e.g., the esophagus or rectum) [5, 8]. Some studies have proposed simulating changes in body temperature by modeling its heat production and dissipation mechanisms [9, 10]. These models divide the body into several nodes and then formulate biomarkers that are correlated with body-temperature regulating mechanisms (e.g., perspiration, blood flow volume), heat transfer and production at various nodes, and heat dissipation to the outside. In this context, estimation precision is proportional to the number of nodes and calculation volume, which results in a mutually exclusive relationship. To accurately estimate body temperature during exercise, it is also important to use estimation methods that are based on temporally variable data. Casa et al. [11] measured body temperature at multiple sites and reported that the oral, axillary, tympanic (aural), and temporal measures were invalid during outdoor exercise in hot environments. In addition, they suggested that rather than using methods for assessing body temperature that may be affected by the environment, skin temperature, sweat, or ingested fluids, other techniques (e.g., assessment of central nervous system function) should be used to measure the temperature of persons who have exercised in a hot environment.

Non-invasive measurement of core body temperature has been a matter of interest over the years, and it is expected to be applied in a wide range of research areas, including sport [12] and medicine [13]. Some simulation models have been proposed to predict heat storage and transfer between physiological indicators and the environment [14, 15]. These models can provide highly accurate results by using multiple indicators, such as environment and clothing. Recently, some studies have attempted to use wearable devices that are designed to sense biodata (e.g., heart rate and body movement) when evaluating physical conditions in hot environments, thus providing a way to predict the risk of heatstroke [16, 17]. The results of these studies have also suggested the feasibility of real-time heat stress evaluations that combine the Internet of Things technology and wearables. Considering practicality, the assessment tool should be non-invasive; furthermore, it should not restrict movement and not involve the placement of a large number of sensors on the body.

To meet these requirements, electrocardiogram (ECG) signals are seen as a useful index for evaluating heatstroke risk. Non-linear analyses and frequency analyses of the R-R interval (RRI) obtained from ECG signals are now widely used and well-known as typical indexes of autonomic nervous system activity [18–20], and can be readily calculated mathematically. Thus, we believe that it would be useful to assess the risk of heatstroke through the decomposition of ECG signals into biomarkers showing various relevant characteristics. The purpose of this study was to propose a useful algorithm designed to estimate core body temperature using ECG signals as a diagnostic criterion for heatstroke. The proposed model estimated continuous core body temperature regardless of individual characteristics such as age and physique, and the validity of the model was verified.

## Materials and methods

### Experimental procedure

The experiment involved an exercise load test, which was performed in an environmental chamber adjusted to a temperature of 35°C and humidity of 50% (Wet Bulb Globe Temperature: WBGT approximately 30°C). Temperature conditions were determined with reference to the average maximum summer temperature (36°C) measured in major cities in Japan over the past 5 years. After ensuring the safety of the participants, the humidity at which the WBGT reaches approximately 30°C (severe warning level) was obtained as a condition of high heat risk using Ono and Tonoishi’s estimation method [21]. The experiment was performed in the following order: rest, 6 min; exercise load, 18 min; rest, 18 min; exercise load, 24 min; rest, 18 min. Physiological signals were measured continuously, and exercise intensity was assessed every 3 min from the start of the experiment using the Borg Rating of Perceived Exertion Scale (RPE) [22]. RPE during exercise was measured by pointing at a score board presented in front of the participant, and the experimenter recorded data every 3 min.

After the experiment, an interview survey was conducted on mood during the experiment (anxiety, tension etc.) and the exercise history of the student. To ensure the safety of the participants, the experiment was immediately discontinued if any of the experiment discontinuation criteria were met (core body temperature exceeding 38.5°C, convulsions, headache, dizziness, nausea/vomiting, blank expression, loss of consciousness, or elevated levels of RPE). A Health Guard II ergometer was used during the test (Takei Scientific Instruments Co., Ltd.). Two conditions with varying exercise loads were used; the ergometer was set to an intensity of 80 W for Condition A and maximum oxygen intake was measured beforehand at 60% for Condition B (where $VO_{2}=3.5+(1.8\times Work\;rate)÷Body\;Weight$, and 1W=6.12×Workrate[kg⋅m∕min]) [23]. The experiment was approved by the Institutional Review Board of the University of Occupational and Environmental Health (Approval No. H29-213) and the study was performed in accordance with the guidelines.

### Participants

The participants included 12 healthy men aged 21–64 years (mean age ± SD = 39.6 ± 13.4) (see Table 1). Six participants who were able to measure maximum oxygen intake performed Condition B in additional to Condition A on a different day. Therefore, 12 participants in Condition A and 6 participants in Condition B performed the test, and a total of 18 test runs were conducted. We preliminarily confirmed that the participants were free of arrhythmia and any diseases related to the cardiovascular system by using a questionnaire containing specific items relating to health examination and verbal communication. The questionnaire comprised items regarding short- and long-term exercise habits. For the item on long-term exercise habits, #10: “Have you been in a habit of exercising to sweat lightly for over 30 min at a time, two times weekly, for over a year?,” seven (58.3%) out of the total 12 participants answered “yes.” For the item on short-term exercise habits, #11: “In your daily life, do you walk or do any equivalent amount of physical activity for more than 1 h a day?,” participants in 12 of the 18 test runs (66.7%) answered “yes.” None of the participants were taking medications. After receiving written descriptions of the experiment, all participants completed written informed consent forms. Further, each participant followed a regulated diet beginning with dinner on the previous day and lasting through breakfast on the test day (alcohol consumption was prohibited). Participants were also instructed not to consume caffeine on the day of the experiment. Further, they were told to coordinate their sleeping environments; this included a bedtime of 23:00 on the day prior and wake time of 6:00 on the day of the experiment.

**Table 1:** Participants and environmental conditions.

No.	Partici-pants	Age	Height (cm)	Weight (kg)	BMI	Exercise Habits		VO2max (ml/kg/min)	Condi-tions	Exercise Load (W)	Status	Note
						#10	#11					
1	S01	57	169	62	21.7	No	Yes	None	A	80	Dis-continued	Poor physical condition, data loss
2	S02	47	175	103	33.6	No	Yes	None	A	80	Completed	
3	S03	46	170	57	19.7	No	Yes	None	A	80	Dis-continued	Rectal temperature reached 38.5°C
4	S04	64	174	70	23.1	No	No	None	A	80	Dis-continued	Poor physical condition
5	S05	53	167	66	23.7	Yes	Yes	None	A	80	Completed	
6	S07	26	184	90	26.6	No	Yes	None	A	80	Completed	
7	S06	31	173	85	28.4	Yes	Yes	44.8	A	80	Completed	Data loss
8						Yes	Yes		B	120	Dis-continued	Poor physical condition
9	S08	21	175	61	19.9	Yes	No	34.6	A	80	Completed	
10						Yes	Yes		B	94	Completed	
11	S09	27	172	80	27.0	Yes	No	62.7	A	80	Completed	
12						Yes	No		B	157	Completed	
13	S10	31	165	67	24.6	No	No	43.3	A	80	Completed	
14						No	No		B	104	Completed	
15	S11	28	167	64	22.9	No	Yes	41.6	A	80	Completed	
16						No	Yes		B	109	Completed	
17	S12	44	184	93	27.5	Yes	Yes	41.3	A	80	Completed	
18						Yes	Yes		B	114	Completed	

BMI, body mass index. The column for exercise habits shows the results of the questionnaire on items regarding specific health examination. Specifically, #10 ("Have you been in a habit of exercising to sweat lightly for over 30 min at a time, 2 times weekly, for over a year?") and #11 ("In your daily life, do you walk or do any equivalent amount of physical activity for more than 1 h a day?”). Participants who performed two test runs under both conditions responded to the exercise habit questions before each test run; thus, these answers are shown separately. In test runs with different exercise intensities, six participants took part in both Conditions A and B; thus, there were 18 test runs. Of these, measurements were discontinued for three cases because the discontinuation criteria were met, while two cases were excluded from analysis due to data loss.

### Physiological measurement

Both pre- and post-experiment, participants were measured for body weight using a precision scale (Combics 1 plus, Sartorius). During the experiment, we continuously measured ECG, core body temperature (rectal temperature), and body surface temperature (chest, palms, outer surface of sensor, inner surface of sensor). ECG was recorded at a 1.5kHz sampling interval with a BSM-2401 wireless electrocardiograph (Nihon Kohden Corporation), while both core body and body surface temperatures were measured with a thermocouple (Class 1 for Type T) and recorded via computer at 10-s intervals. Rectal temperature was measured using a coated thermocouple (3 mm diameter), covered with a disposable probe cover (NIKKISO-THERM CO., LTD.) and Vaseline, 15 cm from the anus, inserted by the participants themselves. A wired flexible cable was connected to ensure stable measurement during exercise. The experiment started after the experimenter confirmed the fixation of the thermocouple and the certainty of the measure values.

### Analysis methods

Regarding precise body weight measurement data, we analyzed differences due to exercise loads through a repeated t-test of the changes. We also calculated differences before and after (Δ = after-before) for each physical data item and investigated the relationship between muscle mass and body height (relative muscle mass). Regarding the analysis of test runs in which participants took part in both conditions (excluding one data loss trial; n = 5), we examined the differences between conditions using a signed test.

Regarding physiological responses, we analyzed 16 total cases, after two (one each in Conditions A and B) were excluded due to data loss (Table 1; S01, S06-A). The continuous measurement data were divided into 28 3-min blocks (Rest1-1~Rest1-2, Ergo1-1~Ergo1-6, Rest2-1~Rest2-6, Ergo2-1~Ergo2-8, and Rest3-1~Rest3-6); we also examined the correlations between various physiological data. Test runs in which the experiment was discontinued were subjected to analysis up to the block at which a 3-min period could be assured. We extracted the RRI from the ECG, then conducted an average of RRI and Poincaré plot analysis in each 3-min block, and heart rate variability (HRV) frequency analysis in each 6-min block. Thus, we calculated six indexes, including average RRI, SD1, SD2, low frequency (LF), high frequency (HF), and LF/HF. For each 3 or 6-min block, we derived core body temperature and the average amount of change in core body temperature $\Delta temp=temp_{\left(i+1\right)}-temp_{i}$, and also examined the correlations between the six indexes. In this regard, we derived the Euclidean distance of each physiological index as converted into a standardized score (z-score) for each participant and examined the degree of similarity in the time series data for both the core body temperature and ECG signals. For the Poincaré plot indices that were strongly correlated with changes in core body temperature, 11 factors (RRI, SD, SD1, SD2, SD1low, SD2low, SD1up, SD2up, SD(i-1), SD1(i-1), SD2(i-1)) were calculated as candidates for independent variables on the basis of mathematical knowledge. The low and up component of each index indicates that these indexes are divided into upper and lower sides with respect to the diagonal of each index. Participants’ age and body mass index (BMI) were added to these 11 indexes, and we deleted variables for which the variance inflation factor (VIF) was 10 or higher. Then, we conducted a multiple regression analysis (forced entry method) wherein the amount of change in core body temperature was treated as the objective variable. IBM SPSS Statistics for Windows, version 19 (IBM corp., Armonk, NY, USA) was used for analysis. Finally, estimate values of this model were evaluated as the agreement with actual measurement values using the Bland-Altman method [24].

A systematic error (bias) is an inaccuracy that has a certain biased tendency toward the true value, and the presence of such systematic errors can be visually verified with the limits of agreement (LoA) method. This method plots the difference (estimated value–actual measured value) against the mean of the actual measured and estimated values, and the LoA are calculated. If the differences between the actual measured and estimated values are within the margin of error, the two values can be interpreted as equivalent.

## Results

### Experiment implementation and changes in physical data

Of the 18 total test runs, four were discontinued (i.e., three participants discontinued—two due to poor physical conditions and one due to elevated rectal temperature; those who discontinued due to poor physical condition were the oldest participants in the study). For test runs in which both conditions were performed, the exercise loads were greater in Condition B. S1 Table shows the results of precise weight measurements before and after the trial. As presented, body weight decreased significantly (rate of change = -1.5%, mean±SE = -1.11±0.09, *p* < .001). The American Conference of Governmental Industrial Hygienists [4] treats a 1.5% body weight reduction (before and after work comparison) as the heat exposure limit value. The rate of weight change found in this study was extremely close to this threshold; thus, we were able to determine that environmental conditions associated with a high risk of heatstroke can be adequately set. In addition, regarding the test runs conducted under both conditions (n = 5), body weight showed a greater reduction tendency in Condition B (mean ± SEM = -0.50 ± 0.14, *p* = .063, sign test).

### Time series changes in physiological responses

**Fig 1:**
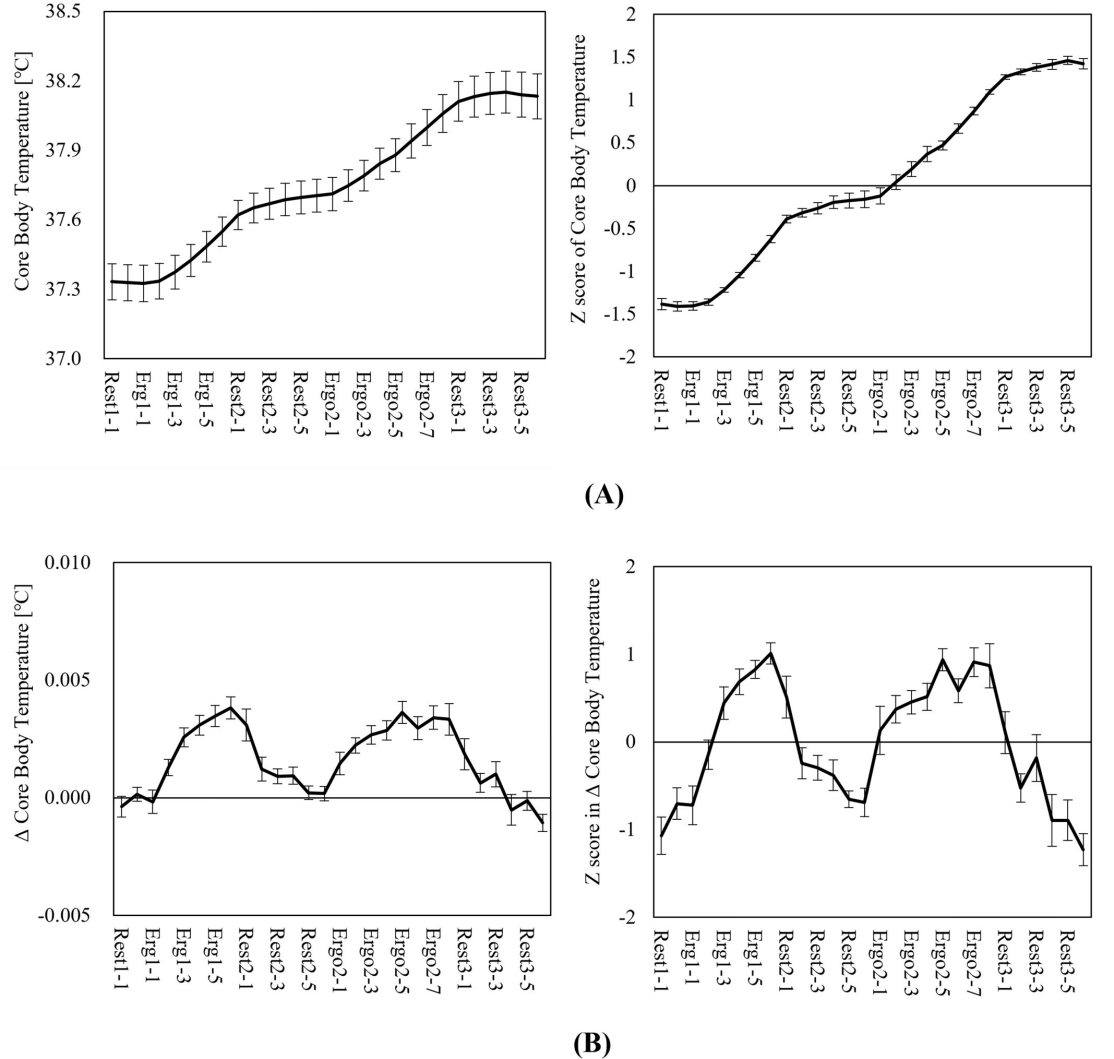
Temporal changes in core body temperature (mean ± SE). (A) Core body temperature. The left side shows the raw data while the right side shows the standardized score (z-score). Core body temperature rose over time, and did not drop, even in the rest block (Rest 2) between exercise blocks. (B) Differences in core body temperature. The left side shows the raw data while the right side shows the standardized score (z-score). No major differences were observed in the rise in body temperature during exercise between the first and second exercise blocks.

**Fig 2:**
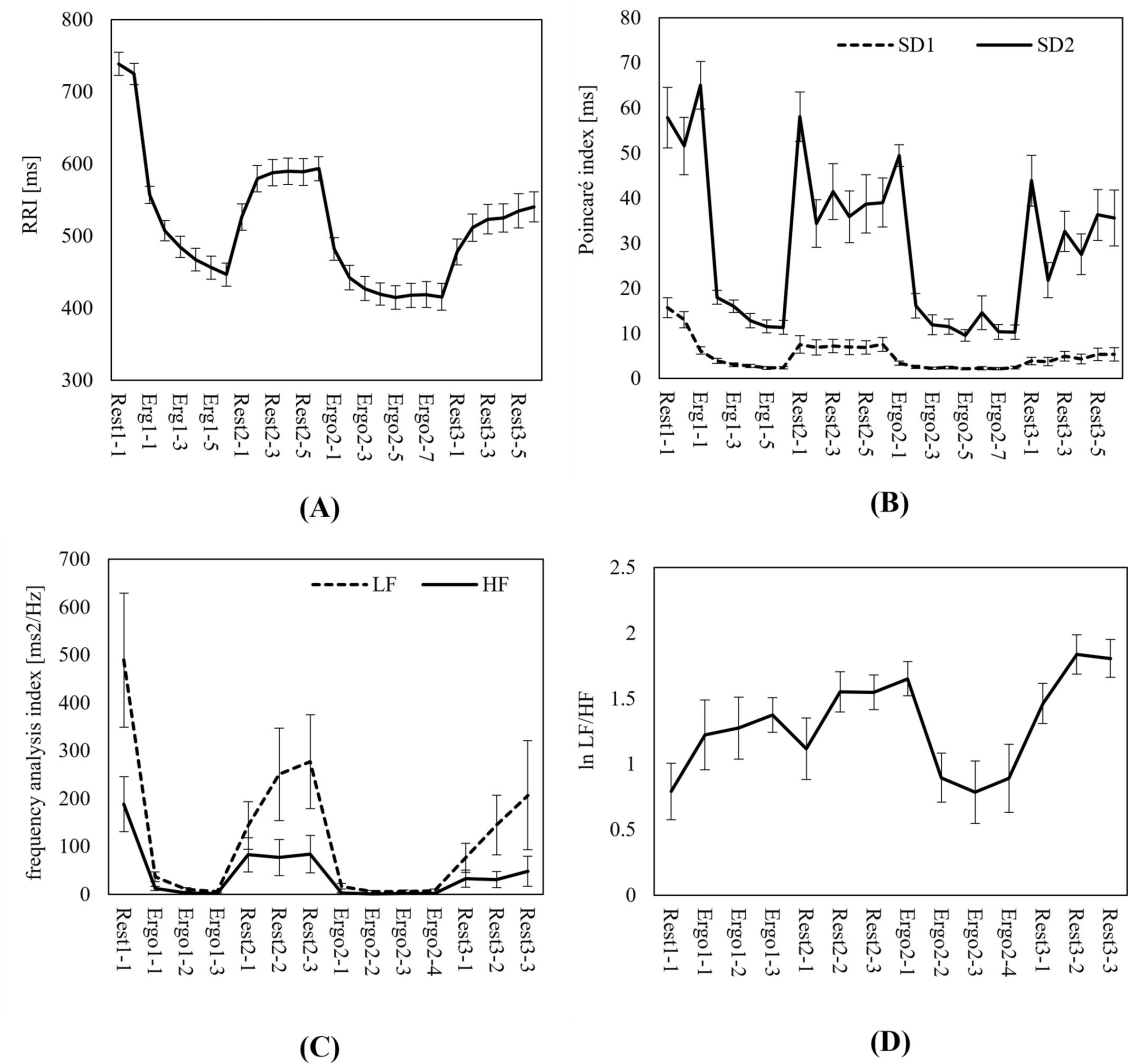
Temporal changes in ECG indexes (mean ± SE). (A) RR interval. The RRI average dropped rapidly according to the exercise load and did not return to the pre-exercise state during rest. (B) Poincaré plot index. SD2 exhibited the same trend as RRI. The change in state from the exercise load to resting state was remarkable. (C, D) Frequency analysis indexes LF/HF. The LF and HF components essentially disappeared during exercise loads, and thus may not be suitable for evaluation under those conditions in the stipulated frequency band.

Fig 1A shows the time series changes in core body temperature, while Fig 1B shows changes in the ΔTemp of core body temperature. Here, core body temperature rose rapidly due to exercise loads, and did not substantially decline during the subsequent 18-min rest period. In particular, a gentle rise continued during the first half of the rest period following the first exercise load (Rest 2); body temperature rose the same degree during the second exercise load as seen at the conclusion of the first exercise load. Approximately the same rises in core body temperature were observed due to the first and second exercise loads. Figs 1B and 2A–2D depict the time series data of the physiological indexes calculated from the ECG signals. Although the RRI average dropped rapidly due to exercise loads and rose during rest, the RRI during rest showed a shortening trend over time without returning to the pre-exercise state during 18 min of rest (Fig 2A). The same trend for RRI was also observed in the Poincaré plot index. SD2 lengthened in blocks with status changes during rest periods following exercise loads, thereby suggesting its possible usefulness in detecting postural and behavioral transformations (Fig 2B). Regarding the HRV indexes for frequency analysis, both the LF and HF components disappeared during exercise loads. Further, it was difficult to ascertain the state during exercise loads within these frequency bands.

After defining the time series data in terms of Eqs (1) and (2), the Euclidean distance (the scale of the similarity measures for the two time series) was easily obtained from Eq (3). The shorter the Euclidean distance, the higher the similarity degree between the two time series data. For comparison of Euclidean distances between all indexes, these were calculated with the standardized score (z-score) for each participant. In the relationship between core body temperature and various physiological indexes, the results of the Euclidean distance measurements (Table 2) clarified changes in ΔTemp with a higher degree of time series data similarity (i.e., rather than core body temperature itself). The Euclidean distances of both the LF and HF components were shorter. However, both indexes disappeared during exercise loads in which the RRI became noticeably short with rapid respiration (see Fig 2C). Hence, these indexes were considered unsuitable as independent variables for estimation of core body temperature. When attempting to estimate core body temperature using ECG signals, these results show the efficacy of setting the amount of change in core body temperature as the objective variable, with the RRI average and Poincaré plot index set as the independent variables.


$$x=(x_{1},x_{2},x_{3},\ldots x_{n})$$ (1)

$$y=(y_{1},y_{2},y_{3},\ldots y_{n})$$ (2)

$$D_{ED}(X,Y)=\sqrt{\sum {\left(y_{i}-x_{i}\right)}^{2}}$$ (3)

**Table 2:** Euclidean distance of core body temperature with indexes from ECG signals.

	Temp	ΔTemp
**RRI**	8.52	4.88
**SD1**	8.46	5.65
**SD2**	7.96	5.49
**LF**	5.57	3.78
**HF**	5.81	3.90
**LF/HF**	4.45	4.56

RRI, R-R intervals. Euclidean distances between various physiological indexes from ECG signals and the Δcore body temperature were shorter than those between these indexes and the core body temperature. That is, a high degree of similarity was exhibited by the Δcore body temperature.

### Multiple regression analysis

**Fig 3:**
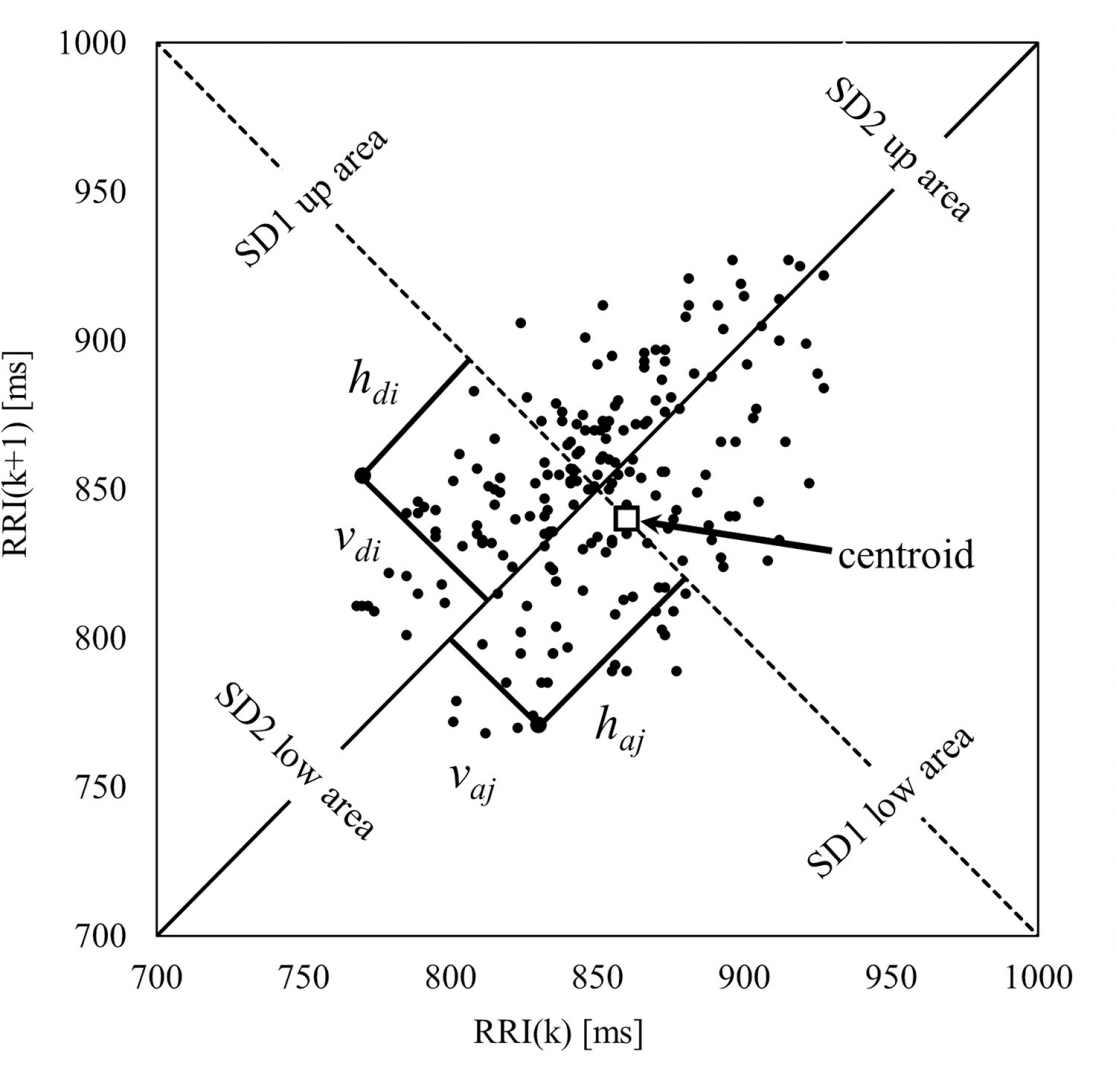
Poincaré plot. The Poincaré plot is defined by the lengths of the short side (SD1) and long side (SD2) of an ellipse drawn by RRI_(k)_ and RRI_(k+1)_. In the RRI, SD1 is an index showing rapid change, while SD2 shows subtle change.

We observed the characteristics of the RRI average and Poincaré plot index, which are believed to be useful in estimating core body temperature. We treated 11 variables from ECG (RRI_mean_, SD, SD1, SD2, SD1low, SD2low, SD1up, SD2up, SD_i_/SD_(i-1)_, SD1_(i)_/SD1_(i-1)_, and SD2_(i)_/SD2_(i-1)_) and two variables from the participants’ characteristics (age and BMI) as candidate independent variables. Poincaré plot indexes were defined by both the standard deviation vertical (SD1) and horizontal (SD2) to the identity line (SD1). Here, SD1low is the lower (origin point) SD1 bifurcated by the diagonal (y = x) in the Poincaré plot coordinates, while SD1up is the length of the top side. SD2low and SD2up are the values at the coordinates shifted by 90° (Fig 3, Eqs (4) and (5)).


$$SD_{1}^{2}=\frac{1}{n}(\sum v_{di}^{2}+\sum v_{ai}^{2})$$ (4)

$$SD_{2}^{2}=\frac{1}{n}(\sum h_{di}^{2}+\sum h_{ai}^{2})$$ (5)

By calculating the multiple covariate index VIF and eliminating variables for which VIF was 10 or higher, we performed a multiple regression analysis of these variables using the forced insertion method, in which we treated the change volume of core body temperature (ΔTemp) as the objective variable. Analysis results showed that the SD2 indexes and BMI remained as significant statistically useful variables to estimate the core body temperature. The adjusted coefficient of determination (adjusted R-squared), which included both the SD2 indexes and BMI, was 0.466. A recalculation of the multiple regression analysis was undertaken as the contribution of BMI was not high. As a result, the adjusted R-squares of the estimation model that had only included the SD2 indexes was only slightly lower, at 0.460 (p < .001). As shown in Table 3, the results suggest that the amount of change in core body temperature ΔTemp(i) may be predicted sufficiently well by the functions SD2(i), SD2(i-1), and ΔTemp(i-1). Eqs (6) and (7), where the symbol Δ denotes the amount of change and ^ the estimated value.


$$\Delta Temp(j)=f(SD_{2}(j),SD_{2}(j-1),\;\hat{T}emp(j-1))$$ (6)

$$\hat{T}emp(j)=Temp(0)+\sum_{i=1}^{j}\Delta Temp(i)$$ (7)

**Table 3:** Multiple regression analysis results.

	Coefficient of Regression	Standard Error	*t*-value	*p*-value
**Intercept**	0.953	0.134	7.13	< .001
**SD2**	-0.078	0.004	-19.37	< .001
**ΔSD2**	0.005	0.001	6.60	< .001
**^Temp**	-0.022	0.004	-6.22	< .001

The amount of change in core body temperature is shown in the SD2, ΔSD2, and previously estimated core body temperature; the adjusted coefficient of determination was R2 = 0.460 (p < .001).

**Fig 4:**
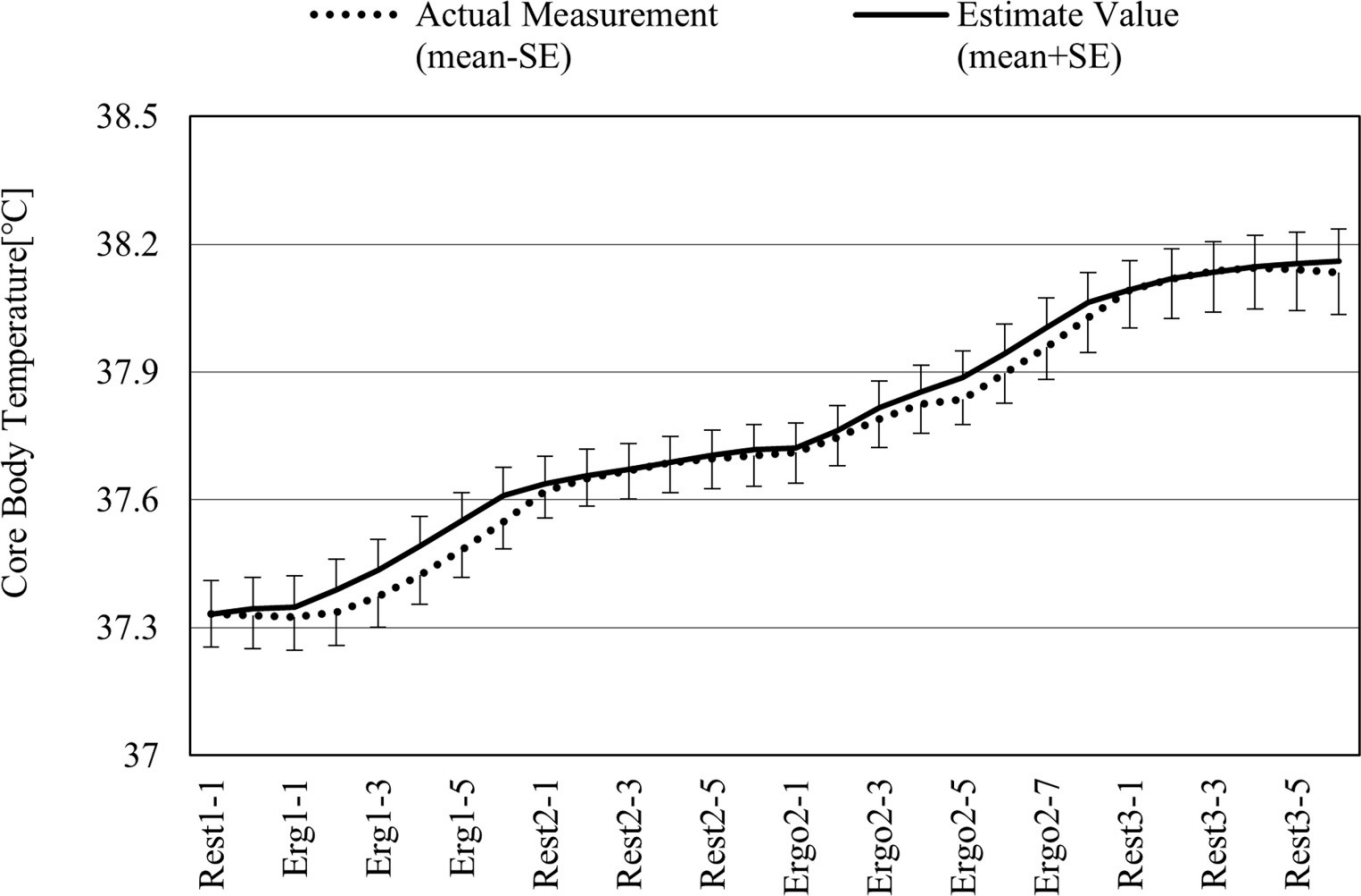
Actual measurements and estimates of core body temperature (average of all test runs). The dotted line shows actual measured values, while the solid line shows estimated values. The mean error was -0.007°C, while the mean error rate was -0.02%. See S1 Fig for actual measurements and estimates from all 16 test runs.

Fig 4 shows the averages for all test runs, including both the actual measurements and estimates of core body temperature. The mean error was -0.007°C, while the mean error rate was -0.02%. The maximum error was 0.457°C in the test run in which the estimated value was largest when compared to the actual measured value (S1A Fig), while the maximum error was -0.445°C in the test run in which the estimated value was the smallest (S1J Fig).

### Estimation model validation

**Fig 5:**
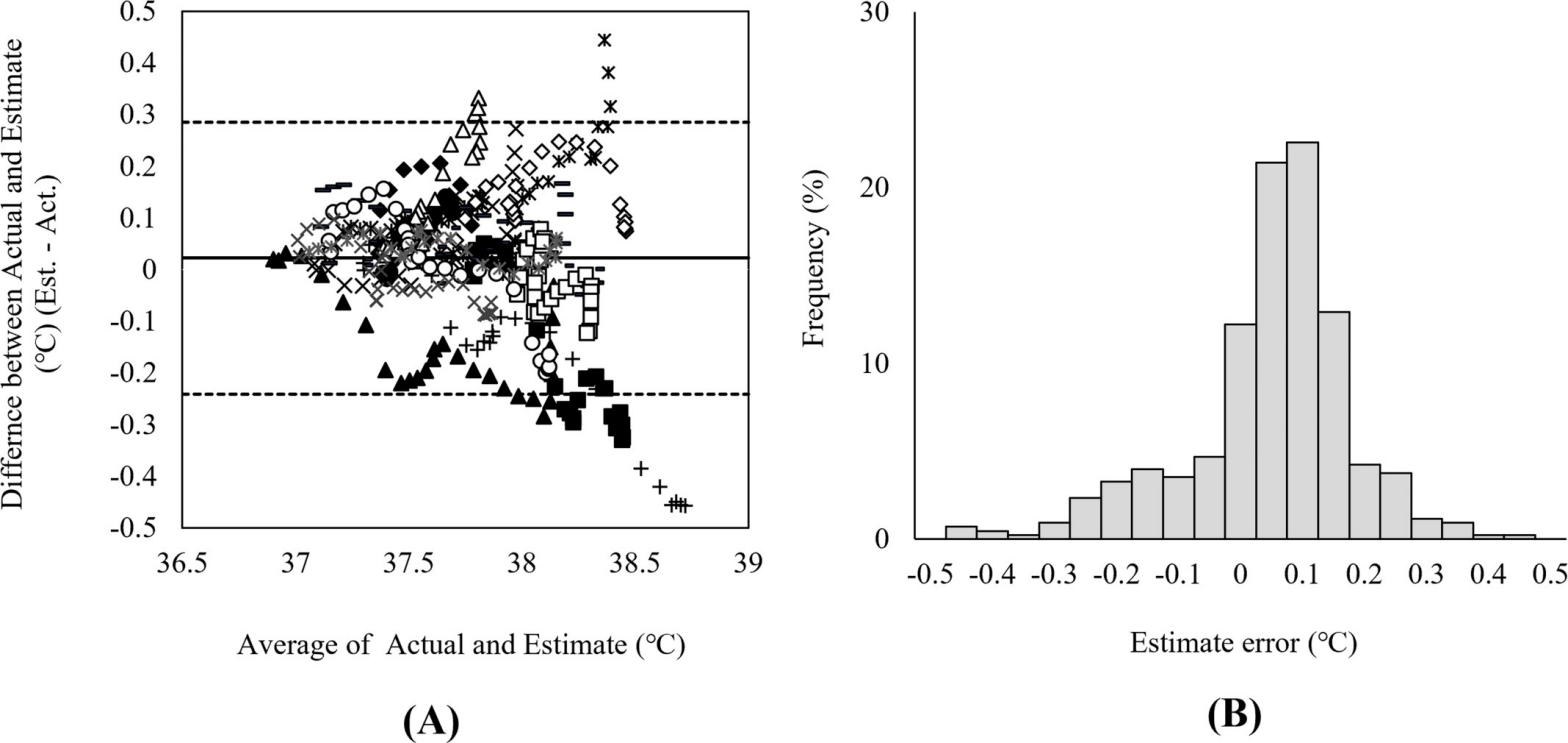
Bland–Altman plot for all participants indicating the bias (solid line) and ±1.96SD (dashed line). (A) Bias ± SD = 0.023 ± 0.13. Different scatter plot markers were used for each participant. (B) Normalized histogram of developed model error for all validation data.

The validity of the model was tested using the Bland-Altman method on 415 estimates of data obtained from the 16 trials. The overall bias was 0.023 ± 0.13°C, and the LoA was ±0.26°C. Fig 5 shows the Bland-Altman plot of the estimation error (estimated–actual measured) against the mean of actual measured value, and the histogram of the estimation error. The results demonstrate that 387 (93.3%) out of 415 points in all trials were within the LoA range, indicating a high agreement rate (Table 4). The Bland-Altman plots and LoA for each participant are shown in S2 Fig.

**Table 4:** Agreement rate against overall LoA range for each participant.

	Within LoA Range	Outside LoA Range		Agreement Rate (%)	Range of Estimation Error
		Act. > Est.	Act. < Est.		
**S02-A**	24	0	3	88.9	-0.01~0.44
**S03-A**	13	11	0	54.2	-0.33~0.05
**S04-A**	16	0	0	100.0	< .001~0.21
**S05-A**	23	4	0	85.2	-0.28~0.03
**S06-A**	24	0	0	100.0	-0.03~0.27
**S07-B**	27	0	0	100.0	0.03~0.14
**S08-A**	27	0	0	100.0	-0.05~0.08
**S08-B**	27	0	0	100.0	0.01~0.17
**S09-A**	27	0	0	100.0	-0.02~0.14
**S09-B**	20	7	0	74.1	-0.46~0.06
**S10-A**	27	0	0	100.0	-0.12~0.08
**S10-B**	27	0	0	100.0	< .001~0.25
**S11-A**	24	0	3	88.9	0.03~0.33
**S11-B**	27	0	0	100.0	-0.20~0.16
**S12-A**	27	0	0	100.0	-0.09~0.10
**S12-B**	27	0	0	100.0	-0.01~0.08
**Total**	387	22	6	93.3	----

The two columns under outside LoA range indicate the negative side (Actual measured values > Estimated values) and positive side (Actual measured values < Estimated values).

## Discussion

First, we discuss the features and advantages of our estimation model in comparison with previous studies. Niedermann et al. [25] suggested that heat flux measurements need to be incorporated to accurately predict rectal temperature. In this context, rectal temperature has been considered by several researchers as the main predictor of core body temperature [8, 26, 27]. Buller et al.’s estimation model for core body temperature using heart rate has been variously validated in studies with a large number of participants [26] and improved versions have been reported [27]. Their model is based on the Kalman filter, which provides a robust estimation algorithm. They have validated this estimation model by considering experimental conditions such as wearing explosive ordnance disposal protective clothing and during recovery [28]. The heat strain decision aid (HSDA) core body temperature prediction model [29] is used for training purposes in the United States military and is currently being improved through multiple studies; specifically, it is being applied to predict safe work continuation times, suitable rest/water intake times, and even the risk of heatstroke, to help develop preventive measures against it. The HSDA includes environmental conditions, clothing, and metabolic heat production as the three important elements mutually attributable to core body temperature; for each participant, rises in core body temperature resulting from exercise are estimated based on individual characteristics (e.g., body height), clothing, the environment (e.g., ambient temperature), and activity status. In this study, demonstrating the close relationship between heart rate and core body temperature quantitatively, we developed a very simple core body temperature estimation model using ECG signals rather than individual characteristics, such as age. Eggenberger et al. [30] verified the validity of two novel multi-parameter models that predict core body temperature during exercise and rest under two clothing conditions, in a hot and moderately humid environment. They found that the "Min-Input Model," which relies on the two most relevant predictors in multiple linear regression analysis, has almost equivalent predictive validity as the "Max-Input Model," which requires six or more input parameters. Their validation suggests that our estimation model for core body temperature using only ECG measurement may provide useful results under other conditions.

Some companies have implemented employee health-management programs that involve monitoring biodata using wearable sensors that use the Internet of Things technologies. This promotes preventive behavior initiated based on real-time warnings in cases where physical conditions are poor and/or the risk of heatstroke is higher. As discussed by Lin et al. [31], however, it is unlikely that verification and proof-of-concept experiments based on physiological characteristics are sufficient for proving the efficacy of such systems. While biometric information is easy to obtain using wearable devices, there are also several limitations in terms of the required resources (e.g., power consumption, calculation power, and memory capacity) and communication status, which cannot always be addressed. In addition, power is needed to accomplish so-called “synchronous” real-time processing, which simultaneously measures multiple biomarkers. Hence, it is important to study estimations that use as few biometrics as possible. From a practical standpoint, the estimation model used in this study is advantageous because of the use of initial core body temperature values and ECG signals without the need for information pertaining to individual characteristics (e.g., the environment or clothing).

In hot environments, the first physical response to heat exposure is dermal vasodilation, which dissipates heat outside the body by increasing the heat transfer rate of the body’s surface through an increase in dermal blood flow. Although this biological response is an efficient and effective response in mildly hot environments, excessive external heat is applied to the body under harsher temperature conditions, thus hindering the heat dissipation function. The blood also tends to accumulate in the extremities because of vasodilation, which causes a drop in both blood pressure and cerebral blood flow, the latter of which may result in dizziness, nausea, and fainting. The body maintains its blood pressure by increasing the heart rate and cardiac output. In other words, heart rate is closely related to the thermoregulatory mechanism. In hot environments, changes in heart rate have been shown to be closely related to changes in rectal temperature [32]. Although it is possible to measure core body temperature directly using ingestible temperature sensors, Hunt et al. [33] confirmed that approximately 10% of them require correction to ensure accuracy. Constantly fluctuating data, such as those pertaining to physical activity volumes, are ideally collected in real time using noninvasive wearable devices, which are suitable for daily use and do not greatly restrict movement. Noninvasive skin, oral, and tympanic temperatures during exercise are easy to measure, but it has been reported that these are strongly affected by two common occurrences, namely, wind and sweat [34]. We indicated that the main disadvantages of using heart rate for the assessment of heatstroke are that it is sensitive to various stimuli, such as mental stress, and that it uses a single criterion when several homeostatic mechanisms are involved simultaneously. However, this is not a major problem, as changes in heart rate due to exercise in a hot environment, such as in our study, are likely to be stronger and longer lasting than changes due to mental stress.

We found that the amount of change in core body temperature, rather than the absolute value, has a stronger relationship with heart rate (RRI). Although its absolute value has been defined as one of the factors for the diagnosis of heatstroke, core body temperature, like normal body temperature, has individual and diurnal variations. Naturally, as heart rate also has individual variations, the risk of error may increase when estimating the absolute value of the core body temperature from heart rate. Consequently, the change in core body temperature or the rate of increase are important factors for managing individual physical conditions. We consider that our estimation model, which calculated the initial value of core body temperature and its changes separately, allows to easily propose various risk factors. If system efficacy is ensured through this proposal, it may be proactively used in high-risk workplaces as an effective countermeasure against heatstroke. Further, the use of various biometric sensors can not only prevent major health disorders resulting from heatstroke but should also facilitate a wide range of other precautionary and health measures, including the prevention of industrial accidents resulting from reduced concentration and increased mental stress, daily health management, and elder care support.

Next, we contend that the selected independent variables in our estimation model are valid, based on psychophysiological knowledge. The Poincaré plot analysis can calculate the nonlinear analysis indexes SD, SD1, and SD2 through a scatter diagram of the adjoining RR intervals. The area of the ellipse valued at the SD (total HRV) correlates with baroreflex sensitivity, LF, HF, and root mean square of successive differences between normal heartbeats (RMSSD). The standard deviation in the short axial direction of the ellipse is referred to as SD1. This index demonstrates the short-term HRV of the rapid RRI change and is the same as the RMSSD [35]. It has been reported that SD1 is related to cardiac vagus nerve function and may be used to indicate exercise intensity (endurance drop) [36]. In contrast, SD2 demonstrates long-term HRV and is considered to be correlated with LF and baroreflex sensitivity [37]. Although reports have shown that *SD*2/*SD*1 and log (*SD*1×*SD*2) are useful indexes of sympathetic and parasympathetic nervous activity, respectively [20], the practical application of these indexes should be assessed both physiologically and mathematically. In this study, the RRI fluctuation was extreme, as it was used as verification in the exercise load test. The SD2 index more noticeably captured RRI fluctuations compared to the amount of change in SD1. However, SD1 remains meaningful in cases where there is little overall change in the RRI (e.g., desk work or light labor) or if the heart rate recovers in a short period of time. Furthermore, the low contribution of BMI to the estimation might indicate that individual characteristic indexes are needed for minor adjustments in relation to individual differences in the RRI, if the RRI value is to be used as an index for estimating core body temperature.

The HRV index obtained by frequency analysis is widely known as an indicator of autonomic nervous system activity, as in the Poincaré plot index. Although some studies have investigated the response to exercise stressor [37, 38], it should be interpreted with caution because the HF component is composed of respiratory sinus arrhythmias derived from respiratory activity. It is important to note that most previous studies using this index have been validated by physiological measurements taken during desk work or in the context of standing up from the supine position, not during exercise [39–41]. Some negative findings regarding this index also exist [42], so there is room for debate about the measurement conditions and other issues. These indexes were also used as candidates for independent variables in this study, but we were able to verify that both the LF and HF components disappeared during exercise loads, during those in which RRI becomes noticeably short with rapid respiration. The HRV components probably shifted to outside the defined frequency bands. While the well-known HRV index based on frequency analysis is now popular in simple stress measurement applications, it is crucial to ensure proper handling based on appropriate mathematical/physiological findings.

The validity of the estimation model was verified by the Bland-Altman method, but the absolute error is also a crucial issue when targeting heatstroke prevention. It is necessary to avoid estimated values that tend to be lower than the actual measurements. Although the allowable range for the core body temperature estimation error is difficult to determine, a limit on the order of 0.5°C is probable if the properties of core body temperature are considered. Examining S09-B (Table 4 and S1J Fig), the largest outlier on the negative side of the LoA range was a participant who was a strong athlete with an exercise intensity of 157 W (on par with hard labor) and maximum oxygen intake of 60%; this participant was evidently accustomed to exercise (as gathered from the interview survey after the experiment). During exercise, the heart rate rises to supply oxygen to the body; however, physical training increases cardiopulmonary function, thus allowing the same exercise to be performed at a lower heart rate. In addition, as post-exercise heart rate recovery (HRR) is correlated with the physical activity Baecke score, HRR is reportedly a useful index for exercise habits [43]. Although a previous study among athletes found a correlation between post-1-min HRR and age [44], it is possible to use a post-3-min recovery index to evaluate exercise adaptability. Considering daily exercise habits, S09 appears to have experienced different HRV and HRR trends than other participants; his Poincaré plot index also exhibited markedly gentle RRI changes. As such, the difference in this heart rate response trend is thought to have caused the estimation error.

Finally, the future practicality of our model must be considered. The periorbital tissue method [45] is innovative and useful in situations where psychological stress is targeted in a space (e.g., a public facility) where a measurement device, such as a video camera, can be fixed. In contrast, the use of wearable devices is essential in situations that do not depend on the place of use, such as workplaces and venues of sports competitions. Negin et al. suggested the possibility of predicting core body temperature from a wearable wrist device in daily life [46]. They noted that this research field is still emerging and undeveloped, and plan to continue their work in the future by expanding the scope of diverse profiles and measurements such as age, gender, health condition, higher body core temperature, and heart rate. Regarding ECG signals, there are many other indexes apart from those investigated in this study [47]. For example, such indexes include those that demonstrate vagus nerve activity in time regions such as pNN50 and the regularity/complexity of time-series data (e.g., approximate entropy and sample entropy) via nonlinear analysis [48–51]. Although these indexes do not necessarily capture all the different types of phenomena, it is possible to identify relationships and differences by contemplating indexes from a mathematical perspective. In other words, it is possible to achieve highly accurate estimation results through a diversified approach that decomposes the same biodata into multiple indexes, which may then be compared based on their unique differences and characteristics. It is also possible to add other physiological responses to phenomena that cannot be captured by ECG signals. Thus, the findings of this study have a wide range of applications, and we plan to conduct additional examinations.

The model we developed in this study has some advantages: it has few errors, is highly practical, and is very simple and easy to understand. Although further validation is required, it contributes to what may be a groundbreaking discovery. Furthermore, although this model is not an immediate substitute for the direct measurement of core body temperature, we believe that it is sufficient to provide an alert of risk in outdoor activities such as heavy labor and sports under hot and humid conditions.

There are several limitations that should be noted in this study. First, the proposed estimation model was targeted at core body temperature increases during exercise loads in hot environments (35°C, 50% humidity); thus, various other environmental conditions remain unverified. In addition, this experiment set a core body temperature exceeding 38.5°C as one of the discontinuation criteria to ensure the safety of the participants, and thus it did not involve extremely high core body temperatures. Therefore, caution should be taken when interpreting the results. Second, the participants wore shorts when biometrics were assessed during the experiment. However, previous investigations using the HSDA model have implemented five types of protective clothing when engaging in treadmill exercises [52]. As differing types of clothing can substantially affect core body temperature, future studies should verify the proposed model under different conditions (e.g., different clothing types and room temperatures). Third, our model requires an initial value of the core body temperature. Although it has been reported that reliable tympanic (aural) measurements are difficult to obtain during outdoor exercise in hot environments [11], it is possible to measure the initial value of core body temperature using tympanic membrane temperature if it is the discontinuous value before exercise [53, 54].

## Conclusions

The most important feature of this study is that it quantitatively demonstrates the close relationship between heart rate and core body temperature, and the results are fitted to a model that estimates rises (amounts of change) in core body temperature during exercise loads in hot environments. In this context, the employed estimation model is characterized by its ability to continuously estimate core body temperature using both its initial value and ECG signals without the need for inputs related to individual characteristics (e.g., age and physique). However, as the estimation error substantially increased in some test runs in the experiment, future investigations should address physical conditions prior to engagement in exercise loads (constituting the initial value) and consider preexisting exercise habits among participants. As our basic estimation model has a wide range of applications, we plan to improve the model for expanded usage (e.g., analyses of cases in which core body temperature drops). In future studies, we also intend to consider the effects of different clothing types and exercise loads, thus demonstrating efficacy across a wider range of applications. We conclude that with the rapid development of technologies such as wearable sensors and the Internet of Things systems, the findings of this study will contribute to healthcare.

## Supporting information

S1 Table Before and after comparison by precise weight measurement. Body weight was significantly reduced. The weight loss of 1.5% or more in 6 of all trials indicated the risk state of heatstroke, according to the American Conference of Governmental Industrial Hygienists [4].
(PDF)

S1 Fig Actual measured and estimated core body temperatures per individual. Dotted lines show actual measured values, while solid lines show estimated values.
(PDF)

S2 Fig Bland-Altman plot for each participant. Solid line and dashed line are indicated bias and ±1.96SD, respectively.
(PDF)

## References

[1] Comparison of the number of people transported to emergency rooms due to heat stroke with the previous year for 2021 (in Japanese). internet. [Cited 2021 December 1]. Available from: https://www.fdma.go.jp/disaster/heatstroke/post3.html#heatstroke02. Japan: Fire and Disaster Management Agency.

[2] Ng CFS, Ueda K, Ono M, Nitta H, Takami A. Characterizing the effect of summer temperature on heatstroke-related emergency ambulance dispatches in the Kanto area of Japan. Int J Biometeorol. 2014;58: 941–948. doi: 10.1007/s00484-013-0677-4 23700200

[3] Bambrick HJ, Dear KBG, Woodruff RE, Hanigan IC, McMichael AJ. The impacts of climate change on three health outcomes: temperature-related mortality and hospitalisations, salmonellosis and other bacterial gastroenteritis, and population at risk from dengue. Garnaut Climate Change Review (S.l.); 2008. 1–47. [Cited 2022 February 1]. Available from: https://eprints.qut.edu.au/103231/.

[4] Am Conference of Governmental Industrial Hygienists. Heat stress and strain. In: 2017; 2017 TLVs and BEIs Based on the Documentation of the Threshold Limit Values for Chemical Substances and Physical Agents & Biological Exposure Indices (Cincinnati, OH: ACGIH). pp. 234–243.

[5] Bouchama A, Knochel JP. Heat stroke. N Engl J Med. 2002;346: 1978–1988. doi: 10.1056/NEJMra011089 12075060

[6] Intergovernmental Panel on Climate Change. The physical science basis. Contribution of working group I to the fifth assessment report of the Intergovernmental Panel on Climate Change. Clim Change. Cambridge: Cambridge University Press. 2013: 2014. doi: 10.1017/CBO9781107415324

[7] International Organization for Standardization. Ergonomics—evaluation of thermal strain by physiological measurements. ISO 9886:2004. Geneva: ISO.

[8] Welles AP, Xu X, Santee WR, Looney DP, Buller MJ, Potter AW, et al. Estimation of core body temperature from skin temperature, heat flux, and heart rate using a Kalman filter. Comput Biol Med. 2018;99: 1–6. doi: 10.1016/j.compbiomed.2018.05.021 29803944

[9] Gagge AP, Stolwijk JAJ, Nishi Y. An effective temperature scale based on a simple model of human physiological regulatory response. ASHRAE Trans. 1971;77: 247–262.

[10] Stolwijk JAJ. A mathematical model of physiological temperature regulation in man. NASA CR-1855. Washington, District of Columbia: National Aeronautics and Space Administration; 1971.

[11] Casa DJ, Becker SM, Ganio MS, Brown CM, Yeargin SW, Roti MW, et al. Validity of devices that assess body temperature during outdoor exercise in the heat. J Athl Train. 2007;42: 333–342. doi: 10.1016/s0162-0908(08)79198-0 18059987 PMC1978469

[12] Armstrong LE, Johnson EC, Casa DJ, Ganio MS, McDermott BP, Yamamoto LM, et al. The American football uniform: Uncompensable heat stress and hyperthermic exhaustion. J Athl Train. 2010;45: 117–127. doi: 10.4085/1062-6050-45.2.117 20210615 PMC2838463

[13] Sessler DI. Temperature monitoring and perioperative thermoregulation. Anesthesiology. 2008;109: 318–338. doi: 10.1097/ALN.0b013e31817f6d76 18648241 PMC2614355

[14] Havenith G. Individualized model of human thermoregulation for the simulation of heat stress response. J Appl Physiol (1985). 2001;90: 1943–1954. doi: 10.1152/jappl.2001.90.5.1943 11299289

[15] Fiala D, Lomas KJ, Stohrer M. Computer prediction of human thermoregulatory and temperature responses to a wide range of environmental conditions. Int J Biometeorol. 2001;45: 143–159. doi: 10.1007/s004840100099 11594634

[16] Runkle JD, Cui C, Fuhrmann C, Stevens S, Del Pinal JD, Sugg MM. Evaluation of wearable sensors for physiologic monitoring of individually experienced temperatures in outdoor workers in southeastern U.S. Environ Int. 2019;129: 229–238. doi: 10.1016/j.envint.2019.05.026 31146157

[17] Buller MJ, Welles AP, Friedl KE. Wearable physiological monitoring for human thermal-work strain optimization. J Appl Physiol (1985). 2018;124: 432–441. doi: 10.1152/japplphysiol.00353.2017 28798200

[18] Shaffer F, McCraty R, Zerr CL. A healthy heart is not a metronome: an integrative review of the heart’s anatomy and heart rate variability. Front Psychol. 2014;5: 1040. doi: 10.3389/fpsyg.2014.01040 25324790 PMC4179748

[19] Piskorski J, Guzik P. Asymmetric properties of long-term and total heart rate variability. Med Biol Eng Comput. 2011;49: 1289–1297. doi: 10.1007/s11517-011-0834-z 21953298 PMC3208812

[20] Toichi M, Sugiura T, Murai T, Sengoku A. A new method of assessing cardiac autonomic function and its comparison with spectral analysis and coefficient of variation of R-R interval. J Auton Nerv Syst. 1997;62: 79–84. doi: 10.1016/s0165-1838(96)00112-9 9021653

[21] Ono M, Tonouchi M. Estimation of wet-bulb globe temperature using generally measured meteorological indices. Jpn J Biometeorol. 2014;50: 147–157. doi: 10.11227/seikisho.50.147

[22] Borg G. Psychophysical scaling with applications in physical work and the perception of exertion. Scand J Work Environ Health. 1990;16 Supplement 1: 55–58. doi: 10.5271/sjweh.1815 2345867

[23] American College of Sports Medicine. General principles of exercise prescription. In: Riebe D, Ehrman JK, Liguori G, Magal M, editors. ACSM’s guidelines for exercise testing and prescription. 10th ed. Zuid-Holland: Wolters Kluwer; 2018. pp. 152–153.

[24] Bland JM, Altman DG. Statistical methods for assessing agreement between two methods of clinical measurement. Lancet. 1986;1: 307–310. doi: 10.1016/s0140-6736(86)90837-8 2868172

[25] Niedermann R, Wyss E, Annaheim S, Psikuta A, Davey S, Rossi RM. Prediction of human core body temperature using non-invasive measurement methods. Int J Biometeorol. 2014;58: 7–15. doi: 10.1007/s00484-013-0687-2 23760405

[26] Buller MJ, Castellani J, Roberts WS, Hoyt RW, Jenkins OC. Human thermoregulatory system state estimation using non-invasive physiological sensors. Annu Int Conf IEEE Eng Med Biol Soc. 2011;2011: 3290–3293. doi: 10.1109/IEMBS.2011.6090893 22255042

[27] Buller MJ, Tharion WJ, Cheuvront SN, Montain SJ, Kenefick RW, Castellani J, et al. Estimation of human core temperature from sequential heart rate observations. Physiol Meas. 2013;34: 781–798. doi: 10.1088/0967-3334/34/7/781 23780514

[28] Hunt AP, Buller MJ, Maley MJ, Costello JT, Stewart IB. Validity of a noninvasive estimation of deep body temperature when wearing personal protective equipment during exercise and recovery. Mil Med Res. 2019;6: 20. doi: 10.1186/s40779-019-0208-7 31196190 PMC6567444

[29] Potter AW, Blanchard LA, Friedl KE, Cadarette BS, Hoyt RW. Mathematical prediction of core body temperature from environment, activity, and clothing: the heat strain decision aid (HSDA). J Therm Biol. 2017;64: 78–85. doi: 10.1016/j.jtherbio.2017.01.003 28166950

[30] Eggenberger P, MacRae BA, Kemp S, Bürgisser M, Rossi RM, Annaheim S. Prediction of core body temperature based on skin temperature, heat flux, and heart rate under different exercise and clothing conditions in the heat in young adult males. Front Physiol. 2018;9: 1780. doi: 10.3389/fphys.2018.01780 30618795 PMC6295644

[31] Lin SS, Lan CW, Hsu HY, Chen ST. Data analytics of a wearable device for heat stroke detection. Sensors (Basel). 2018;18: 4347. doi: 10.3390/s18124347 30544887 PMC6308959

[32] World Health Organization. Health factors involved in working under conditions of heat stress. report of a WHO scientific group. internet. World Health Organ Tech Rep Ser. Meeting held in Geneva from 29 August to 4 September. 1969. [Cited 2021 November 25]. Available from: https://apps.who.int/iris/handle/10665/40716;412: 1–32. 4979147

[33] Hunt AP, Bach AJE, Borg DN, Costello JT, Stewart IB. The systematic bias of ingestible core temperature sensors requires a correction by linear regression. Front Physiol. 2017;8: 260. doi: 10.3389/fphys.2017.00260 28496414 PMC5406512

[34] Morán-Navarro R, Courel-Ibáñez J, Martínez-Cava A, Conesa-Ros E, Sánchez-Pay A, Mora-Rodriguez R, et al. Validity of skin, oral and tympanic temperatures During exercise in the heat: effects of wind and sweat. Ann Biomed Eng. 2019;47: 317–331. doi: 10.1007/s10439-018-02115-x 30136150

[35] Ciccone AB, Siedlik JA, Wecht JM, Deckert JA, Nguyen ND, Weir JP. Reminder: RMSSD and SD1 are identical heart rate variability metrics. Muscle Nerve. 2017;56: 674–678. doi: 10.1002/mus.25573 28073153

[36] Tulppo MP, Mäkikallio TH, Seppänen T, Laukkanen RT, Huikuri HV. Vagal modulation of heart rate during exercise: effects of age and physical fitness. Am J Physiol. 1998;274: H424–H429. doi: 10.1152/ajpheart.1998.274.2.H424 9486244

[37] Cottin F, Leprêtre PM, Lopes P, Papelier Y, Médigue C, Billat V. Assessment of ventilatory thresholds from heart rate variability in well-trained subjects during cycling. Int J Sports Med. 2006;27: 959–967. doi: 10.1055/s-2006-923849 17190003

[38] Karapetian GK, Engels HJ, Gretebeck RJ. Use of heart rate variability to estimate LT and VT. Int J Sports Med. 2008;29: 652–657. doi: 10.1055/s-2007-989423 18213538

[39] Castrillón CIM, Miranda RAT, Cabral-Santos C, Vanzella LM, Rodrigues B, VanderLei LCM, et al. High-intensity intermittent exercise and autonomic modulation: effects of different volume sessions. Int J Sports Med. 2017;38: 468–472. doi: 10.1055/s-0042-121898 28388782

[40] Montano N, Ruscone TG, Porta A, Lombardi F, Pagani M, Malliani A. Power spectrum analysis of heart rate variability to assess the changes in sympathovagal balance during graded orthostatic tilt. Circulation. 1994;90: 1826–1831. doi: 10.1161/01.cir.90.4.1826 7923668

[41] Pagani M, Lombardi F, Guzzetti S, Rimoldi O, Furlan R, Pizzinelli P, et al. Power spectral analysis of heart rate and arterial pressure variabilities as a marker of sympatho-vagal interaction in man and conscious dog. Circ Res. 1986;59: 178–193. doi: 10.1161/01.res.59.2.178 2874900

[42] Billman GE. The LF/HF ratio does not accurately measure cardiac sympatho-vagal balance. Front Physiol. 2013;4: 26. doi: 10.3389/fphys.2013.00026 23431279 PMC3576706

[43] Buchheit M, Gindre C. Cardiac parasympathetic regulation: respective associations with cardiorespiratory fitness and training load. Am J Physiol Heart Circ Physiol. 2006;291: H451–H458. doi: 10.1152/ajpheart.00008.2006 16501030

[44] Suzic Lazic J, Dekleva M, Soldatovic I, Leischik R, Suzic S, Radovanovic D, et al. Heart rate recovery in elite athletes: the impact of age and exercise capacity. Clin Physiol Funct Imaging. 2017;37: 117–123. doi: 10.1111/cpf.12271 26147945

[45] Shastri D, Tsiamyrtzis P, Pavlidis I. Periorbital thermal signal extraction and applications. Annu Int Conf IEEE Eng Med Biol Soc. 2008;2008: 102–105. doi: 10.1109/IEMBS.2008.4649101 19162604

[46] Nazarian N, Liu S, Kohler M, Lee JKW, Miller C, Chow WTL, et al. Project Coolbit: can your watch predict heat stress and thermal comfort sensation? Environ Res Lett. 2021;16. doi: 10.1088/1748-9326/abd130

[47] Shaffer F, Ginsberg JP. An overview of heart rate variability metrics and norms. Front Public Health. 2017;5: 258. doi: 10.3389/fpubh.2017.00258 29034226 PMC5624990

[48] Beckers F, Ramaekers D, Aubert AE. Approximate entropy of heart rate variability: validation of methods and application in heart failure. Cardiovasc Eng. 2001;1: 177–182. doi: 10.1023/A:1015212328405

[49] Pincus SM. Approximate entropy as a measure of system complexity. Proc Natl Acad Sci U S A. 1991;88: 2297–2301. doi: 10.1073/pnas.88.6.2297 11607165 PMC51218

[50] Richman JS, Moorman JR. Physiological time-series analysis using approximate entropy and sample entropy. Am J Physiol Heart Circ Physiol. 2000;278: H2039–H2049. doi: 10.1152/ajpheart.2000.278.6.H2039 10843903

[51] Umetani K, Singer DH, McCraty R, Atkinson MC. Twenty-four hour time domain heart rate variability and heart rate: relations to age and gender over nine decades. J Am Coll Cardiol. 1998;31: 593–601. doi: 10.1016/s0735-1097(97)00554-8 9502641

[52] Wang F, Gao C, Kuklane K, Holmér I. Effects of various protective clothing and thermal environments on heat strain of unacclimated men: the PHS (predicted heat strain) model revisited. Ind Health. 2013;51: 266–274. doi: 10.2486/indhealth.2012-0073 23385435

[53] Yeoh WK, Lee JKW, Lim HY, Gan CW, Liang W, Tan KK. Re-visiting the tympanic membrane vicinity as core body temperature measurement site. PLOS ONE. 2017;12: e0174120. doi: 10.1371/journal.pone.0174120 28414722 PMC5393563

[54] Childs C, Harrison R, Hodkinson C. Tympanic membrane temperature as a measure of core temperature. Arch Dis Child. 1999;80: 262–266. doi: 10.1136/adc.80.3.262 10325708 PMC1717865

