# Supplementary material for: Estimating core body temperature using electrocardiogram signals
Source: PLoS One. 2022 Jun 28;17(6):e0270626. doi: 10.1371/journal.pone.0270626 (PMC9239487; doi:10.1371/journal.pone.0270626)
Supplement: S1 Table — Body weight was significantly reduced. The weight loss of 1.5% or more in 6 of all trials indicated the risk state of heatstroke, according to the American Conference of Governmental Industrial Hygienists [4]. (PDF) [file pone.0270626.s001.pdf]

S1 Table. Before and After Comparison by Precise Weight Measurement.

Trial	Before (kg)	After	Before-after	Change Percent (%)
S02-A	103.196	102.390	-0.81	-0.78
S03-A	56.904	56.027	-0.88	**-1.54**
S04-A	69.749	69.071	-0.68	-0.97
S05-A	65.437	64.541	-0.90	-1.37
S06-B	85.404	84.399	-1.01	-1.18
S07-A	89.927	88.824	-1.10	-1.23
S08-A	60.980	60.177	-0.80	-1.32
S08-B	59.597	58.373	-1.22	**-2.05**
S09-A	79.617	78.758	-0.86	-1.08
S09-B	79.085	77.233	-1.85	**-2.34**
S10-A	66.863	66.041	-0.82	-1.23
S10-B	68.118	67.169	-0.95	-1.39
S11-A	63.823	62.660	-1.16	**-1.82**
S11-B	66.265	64.609	-1.66	**-2.50**
S12-A	93.196	91.934	-1.26	-1.35
S12-B	94.090	92.364	-1.73	**-1.83**
mean±SE	75.14±3.57	74.04±3.55	-1.11±0.09	**-1.50**

Body weight was significantly reduced. The weight loss of 1.5% or more in 6 of all trials indicated the risk state of heatstroke, according to the American Conference of Governmental Industrial Hygienists [4].
