# Supplementary material for: Estimating core body temperature using electrocardiogram signals
Source: PLoS One. 2022 Jun 28;17(6):e0270626. doi: 10.1371/journal.pone.0270626 (PMC9239487; doi:10.1371/journal.pone.0270626)
Supplement: S1 Fig — Dotted lines show actual measured values, while solid lines show estimated values. (PDF) [file pone.0270626.s002.pdf]

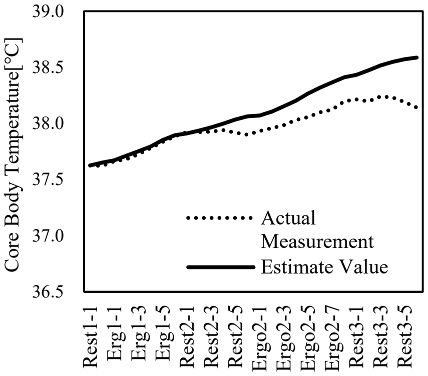

(A) S02-A

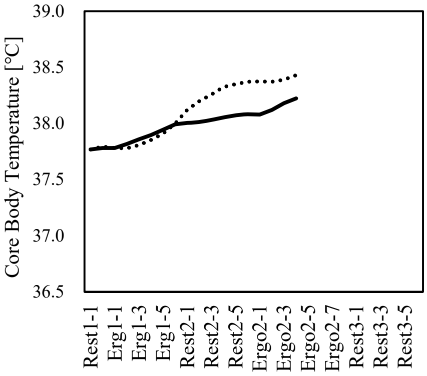

(B) S03-A

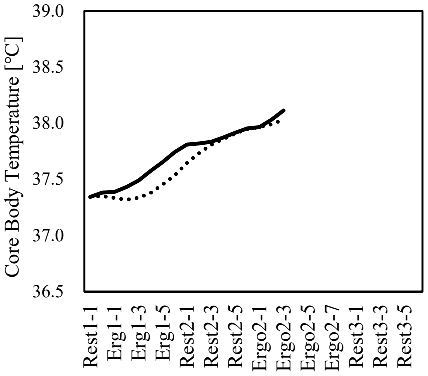

(C) S04-A

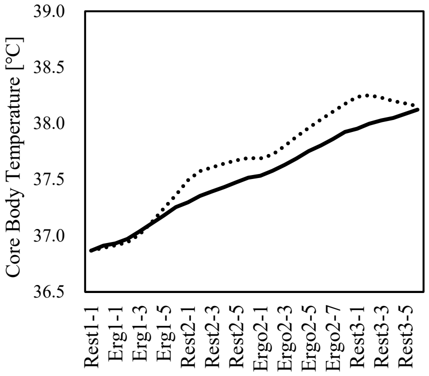

(D) S05-A

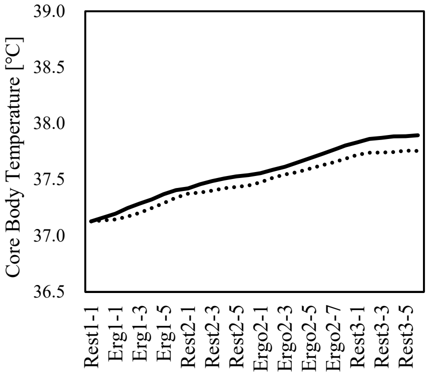

(E) S06-A

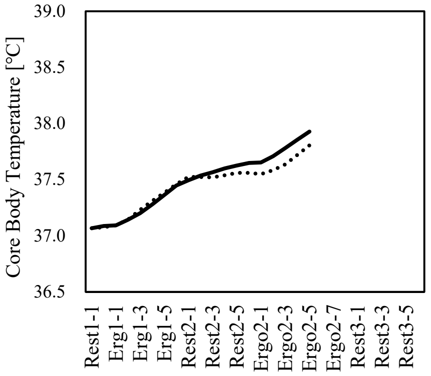

(F) S07-B

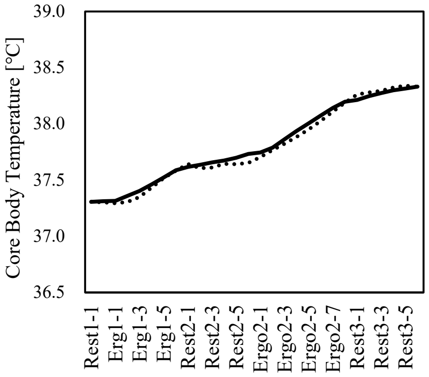

(G) S08-A

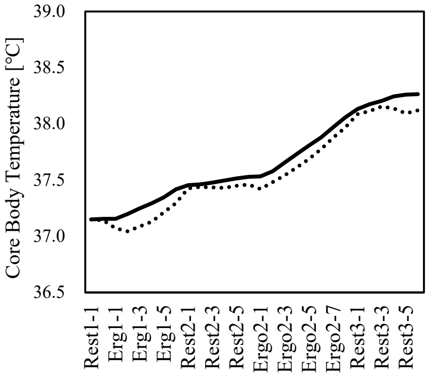

(H) S08-B

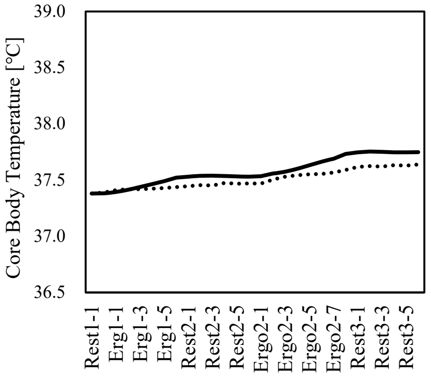

(I) S09-A

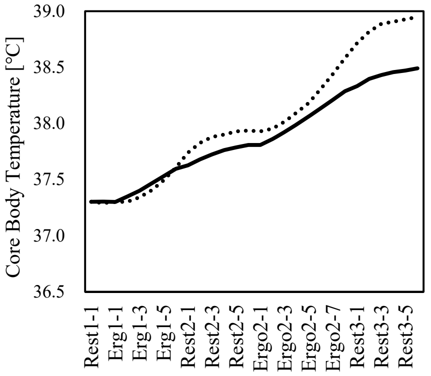

(J) S09-B

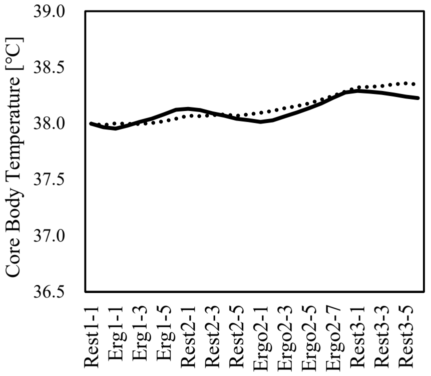

(K) S10-A

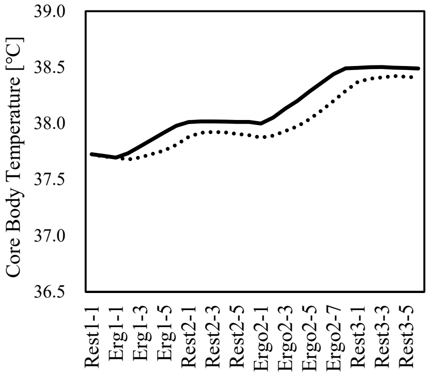

(L) S10-B

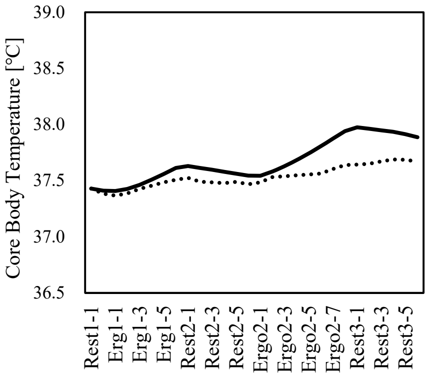

(M) S11-A

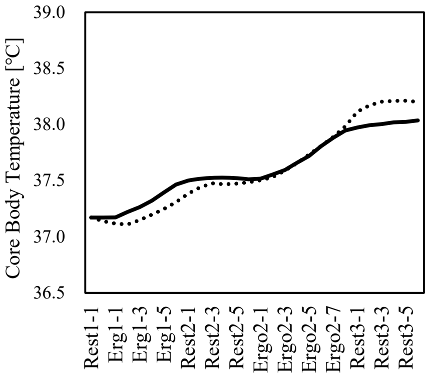

(N) S11-B

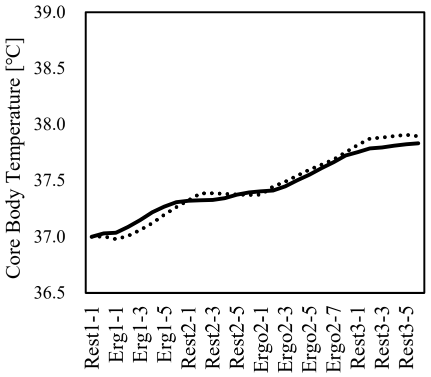

(O) S12-A

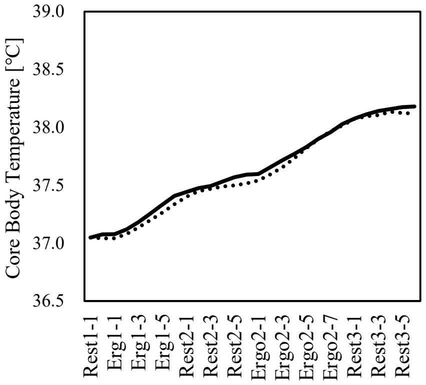

(P) S12-B

S2 Fig. Actual measured and estimated core body temperatures per individual. Dotted lines show actual measured values, while solid lines show estimated values.
